# Inhibitory effects of 2-methacryloyloxyethyl phosphorylcholine polymer on the adherence of bacteria causing upper respiratory tract infection

**DOI:** 10.1080/20002297.2020.1808425

**Published:** 2020-08-20

**Authors:** Hiroyuki Iuchi, Junichiro Ohori, Takayuki Kyutoku, Kotoko Ito, Masaki Kawabata

**Affiliations:** Department of Otolaryngology, Head and Neck Surgery, Kagoshima University Graduate School of Medical and Dental Sciences, Kagoshima, Japan

**Keywords:** 2-Methacryloyloxyethyl phosphorylcholine, nontypeable *Haemophilus influenza*, *streptococcuspneumonia*, phosphorylcholine, inhibitory effect, detroit 562 cells, balb/c mice

## Abstract

**Objective:**

We aimed to investigate the inhibitory effect of 2-methacryloyloxyethyl phosphorylcholine (MPC) polymer on the adherence of *Streptococcus pneumoniae* (Spn) and nontypeable *Haemophilus influenzae* (NTHi) *in vitro* and *in vivo*.

**Materials and Methods:**

Phosphorylcholine (PC) expression of 21 strains each of Spn and NTHi was evaluated using fluorescence-activated cell sorting; the adherence of bacteria to Detroit 562 cells and to the nasal mucosa of BALB/c mice was determined. MPC polymer-mediated inhibitory effects were compared with PC-keyhole limpet hemocyanin (PC-KLH)-mediated inhibitory effects.

**Results:**

*In vitro* experiments showed that pretreatment with MPC polymer markedly inhibited the adherence of Spn and NTHi in a concentration dose–dependent manner independently of PC expression. No correlation was observed between PC expression and MPC polymer-mediated inhibitory effects. Contrarily, there was a significant negative correlation between PC-KLH-mediated inhibitory effects and PC expression in Spn and NTHi. The same results were obtained via *in vivo* experiments. The MPC polymer did not affect the histology of the nasal mucosa.

**Conclusions:**

MPC polymer might be effective to reduce the occurrence of upper respiratory tract infection caused by Spn and NTHi and could be applied for the development of local treatments, such as topical gargles and nebulizer medications.

## Introduction

*Streptococcus pneumoniae* (Spn) and nontypeable *Haemophilus influenzae* (NTHi) are major pathogens in acute upper respiratory tract infections, such as acute otitis media, acute sinusitis, and acute epiglottitis [1,2]. Despite the continued development of antibacterial agents that are excellent treatment options, multidrug-resistant bacteria are increasing year by year, and severe cases that cannot be controlled with oral antibacterial agents are often encountered [3].To prevent these infections, pneumococcal and type B*H.influenzae* vaccines are routinely administered, but because these vaccines are ineffective against nonvaccine strains and NTHi, they have little effect against upper respiratory tract infections [4,5]. Gargle and nebulizer therapiesare commonly used for preventing and treating upper respiratory tract infections. Povidoneiodine–containing water has a bactericidal effect on oral bacteria [6], and nebulizer therapy with antibiotics is effective for sinusitis [7]. However, the effects of gargles are limited [8], and only cefmenoxime, a third-generation cephalosporin, is currently indicated for use in nebulizer therapy [9]. Development of other topical medications to be used in gargle and nebulizer therapies to prevent upper respiratory tract infections is expected. One of the candidates is 2-methacryloyloxyethyl phosphorylcholine (MPC).

This phosphorylcholine (PC)-containing MPC polymer possesses phospholipid polar group sidechains, which provide a highly hydrophilic surface that can resist protein adsorption and bacterial adherence [10,11]. For instance, coating surfaces with MPC polymer has been found to reduce the adherence of *Escherichia coli* [10]. MPC polymer can be used to control oral microbiotatoprevent further oral infection, including dental caries and periodontitis [12]. However, the precise mechanism by which MPC polymer inhibits bacterial adherence, as well as the effects of MPC polymer on Spn and NTHi, remain unknown. Many factors are associated with the adherence of Spn and NTHi, and PC is one of the adherence factors in these bacteria.

PC is a structural component of various pathogens, such as Spn and NTHi [13]. A monoclonal anti-PC antibody (TEPC-15) was found to inhibit the adherence of PC-expressing bacteria by blocking platelet-activating factor receptor (PAF-R) [14]. We reported that the adherence of Spn and NTHi and the invasion of cultured human epithelial cells by NTHi were significantly reduced in the PC-high group by pretreatment with a PC-keyhole limpet hemocyanin (PC-KLH) [15]. Cundell et al. investigated the attachment of bacterial PC to PAF-R and found that binding between PC and PAF-R enhanced the adherence of Spn and only virulent Spn engaged in PAF-R [14]. In other words, it is considered that the inhibition of PAF-R with PC-KLH can inhibit the adhesion of highly pathogenic bacteria to cells.

In the present study, we investigated the inhibitory effect of MPC polymer on the adherence of Spn and NTHi, and we compared the inhibitory effects of MPC polymer with those of PC-KLH on the adherence of Spn and NTHi to clarify the association of PC expression with the effects of MPC polymer.

## Materials and methods

### Bacteria and growth conditions

We evaluated fourlaboratory strains of Spn (EF3030, TIGR4, D39, and L82016; provided by Professor Hotomi, University of Wakayama, Wakayama, Japan); 13 clinical strains of Spn and 18 clinical strains of NTHi isolated from the nasopharynx of patients with otitis media with effusion; two clinical strains of Spn and three clinical strains of NTHi isolated from the blood and spinal fluid of patients with meningitis (provided by Professor Nishi, University of Kagoshima, Kagoshima, Japan); and two American Type Culture Collection (ATCC) strains of Spn (6303 and 6312). All bacteria were stored in skimmed milk with glycerol at −80°C until use. An aliquot of each bacterial stock was thawed and cultured overnight at 37°C in a 5% CO_2_ incubator on sheep blood agar (Nissui Pharmaceutical Co., Ltd., Tokyo, Japan) or chocolate II agar (Nippon Becton Dickinson Co. Ltd., Tokyo, Japan) plates, as appropriate. After washing in 0.5% bovine serum albumin-phosphate-buffered saline (PBS), the bacteria were used for cell adherence assays. The concentrations of Spn and NTHi were adjusted to 1.0 × 10^8^ colony-forming units (CFU)/mL at an absorbance of 580 nm.

### MPC polymer

MPC copolymerizes efficiently with styrene and with alkyl methacrylate such as butyl methacrylate (BMA). Poly [MPC-*co*-BMA] [PMB] was used. As PMB, 5.0 wt% aqueous solution (Lipidure-PMB, NOF Corporation, Tokyo, Japan) with an MP/BMA copolymerization composition ratio of 8:2 was used. The molecular weight of the polymer was >5 × 10^5^.

### Detroit 562 cellculture

Detroit 562 cells (CCL-138; ATCC, Manassas, VA), a human pharyngeal carcinoma epithelial cell line, were grown to confluence in minimal essential medium (Nacalai Tesque Inc., Kyoto, Japan) supplemented with 1 mM sodium pyruvate (Nacalai Tesque), 10% fetal bovine serum (Invitrogen, San Diego, CA), penicillin (100 U/mL), and streptomycin (100 μg/mL; Nacalai Tesque) at 37°C in a 5%CO_2_ incubator. The cells were then harvested using trypsin (final concentration, 0.02%) and ethylenediaminetetraacetic acid (EDTA; final concentration, 0.02%; Nacalai Tesque) and seeded at a density of 2 × 10^4^ viable cells per well in a 96-well BD Falcon tissue culture plate with a low evaporation lid (BD Biosciences, Franklin Lakes, NJ). The plates were used when >90% confluence was observed following overnight incubation.

### Mice

Six-week-old female BALB/c mice were obtained from CLEA Japan (Shizuoka, Japan) and maintained in the experimental animal facility of Kagoshima University under specificpathogen-free conditions. The experimentswere performed when the mice were 7 weeks old. The experimental protocol was approved by the Ethics Board of the Institute of Laboratory Animal Sciences of Kagoshima University (approval numbers 16,110 and 17,110).

#### Adherence of MPC polymer to detroit 562cells

Fluorescence-activated cell sorting (FACS) was performed on a CytoFLEX benchtop flow cytometer(Beckman Coulter, Tokyo, Japan) to determine the adherence of MPC polymerto epithelial cells. We obtained 5% MPC polymer (Lipidure®) and 5% fluorescein isothiocyanate (FITC)-labeled MPC polymer from NOF Corporation (Tokyo, Japan). The cells were incubated for 30 min at 25°C with the FITC-labeled MPC polymer. Next, the mean fluorescence intensity (MFI) of the MPC polymerwas measured. We also analyzedthe micrographs of the MPC polymer on the MPC-treated cultured cells using a BZ-X710 All-in-One Fluorescence Microscope (Keyence, Osaka, Japan). Immunofluorescence images of each well were taken to visualize the FITC-labeled MPC polymer signals (1-s exposure, 488 nm) at 200× magnification.

#### PC expression of Spn and NTHi

The levels of PC expression on bacterial surfaces were quantified via FACS using a CytoFLEX flow cytometer. Bacteria that had been cultured overnight on blood agar or chocolate II agar plates, as appropriate, were suspended in PBS and incubated at 4°C for 4 h with TEPC-15 (Sigma-Aldrich, St. Louis, MO), which is a PC-specific monoclonal immunoglobulin (Ig) A antibody (1:100 dilution), or with a purified mouse IgA antibody (1:50 dilution; BD Biosciences) as an isotype control. Next, the bacteria were rinsed in PBS and incubated with an FITC-labeled goat anti-mouse antibody (1:50 dilution; KPL, Gaithersburg, MD) for 30 min at 20°C before analysis.

#### Adherence assay (*in vitro*)

Detroit 562 cells were adhered to the wells of a 96-well plate (Thermo Fisher Scientific, Nunc A/S, Roskilde, Denmark). Next, we added 100 μL of each bacterial strain (Spn: 1.0 × 10^7^CFU/mL and NTHi: 1.0 × 10^5^CFU/mL) to the cells and allowed them to adhere at 37°C in a 5% CO_2_ incubator for 2 h. Next, we washed each well 10 times with 200 μL of PBS and treated each well with 100 μL of saponin at 37°C in a 5%CO_2_ incubator for 15 min. Finally, we plated 100 μL of the solution from each well on sheep blood agar or chocolate II agar plates, as appropriate, and evaluated the numbers of colonies after 12 h of incubation. To investigate the effects of MPC polymer on bacterial adherence, epithelial cells that had been previously treated with 3.5% MPC polymer at 37°C in a 5%CO_2_ incubator for 1 h were subjected to an adherence assay. The numbers of adherent bacteria of each strain were counted by two observers who were blinded to the experimental conditions.The experiment was repeated four times for each bacterium.

#### *Adherence assay (*in vivo)

To examine the adherence of Spn and NTHi *in vivo*, the mice were pretreated intranasally with 10 μL of MPC polymer (0.5%) or PC-KLH (100 μg/mL) for 1 h. Next, 10 μL of Spn or NTHi suspended in PBS (1.0 × 10^6^CFU/mL) was administered. The mice were sacrificed 12 h after bacterial inoculation, and the nasal passages were removed. The nasal passage samples were homogenized in 1 mL of culture medium. The samples were spread on agar plates, similar to the nasal wash samples. Finally, the number of colonies was counted following overnight incubation at 37°C with 5% CO_2_. The numbers of adherent bacteria of each strain were counted by two observers blinded to the experimental conditions. The experiment was repeated four times for each bacterium.

#### Effect of MPC polymer on the nasal mucosa

We examined the effect of MPC polymer on the nasal mucosa by surgically removing the nasal passage tissues from themice after pretreatment for 12 h with 0.5% MPC polymer or PBS (control). We fixed the tissues in 4% paraformaldehyde for 16 h at 4°C. The fixed tissues were decalcified in EDTA solution for 10 days at 4°C and embedded in paraffin blocks. The samples were sliced into 5-μm-thick coronal sections, which were stained with hematoxylin and eosin. Investigators who were blinded to the previous treatments measured the thickness of the nasal mucosa in the nasal septum by high-magnification (200×) microscopy.

### Statistical analysis

All values were presented as means ± standard deviation. The data were statistically analyzed using the unpaired one-way ANOVA with Tukey’s method and Pearson’s correlation coefficient (cross-reaction data).We considered differences to be statistically significant when the probability values were <5%.

## Results

### Adherence of MPC polymer to cultured cells

**Figure 1.**
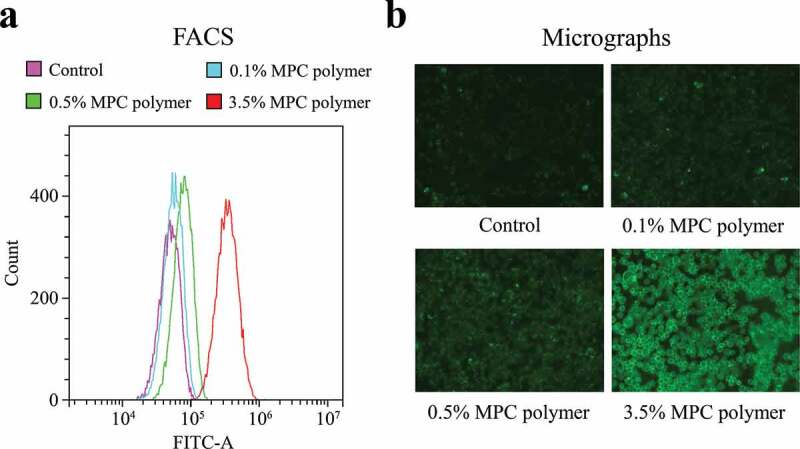
Adherence of MPC polymer to cultured cells.

The MFI values increased in a concentration-dependent manner, indicating that the higher the concentration of MPC polymer, the more MPC polymer adhered to Detroit 562 cells (Figure 1(a)). The concentration-dependent adherence of MPC polymer was confirmed by immunofluorescence micrographic examination (Figure 1(b)).

Adherence of 2-methacryloyloxyethyl phosphorylcholine (MPC) polymer to Detroit 562 cells was measured by fluorescence-activated cell sorting (FACS) (a) and micrographs (b). Cells treated with phosphate-buffered saline (PBS) were used as the control. Compared with controls, the adherence of MPC polymer to the cells increased with increasing concentrations of MPC polymer of 0.1%, 0.5%, and 3.5%. FITC-A, fluoresce in isothiocyanate A.

### Inhibitory effects of MPC polymer on bacterial adherencein vitro

**Figure 2.**
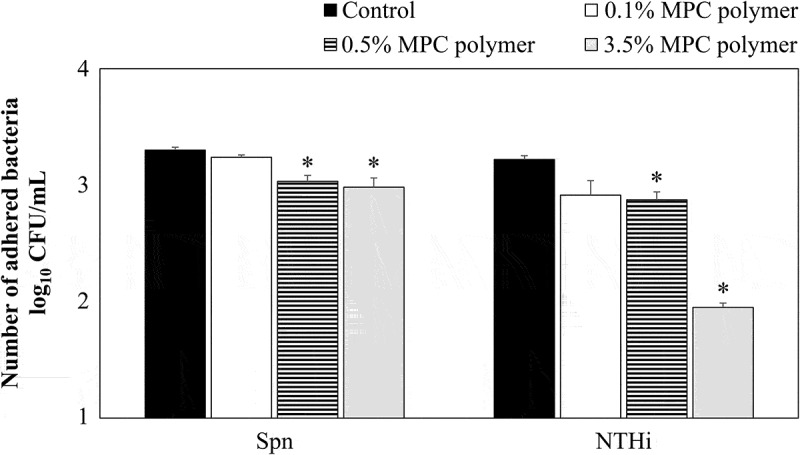
Effects of MPC polymer concentration on the adherence of Spn and NTHi *in vitro.*

Because cells detached from the plate when they were treated with the 5% MPC polymer, 3.5% MPC polymer was used as the maximum concentration for*in vitro* experiments. As shown in Figure 2, the number of adhered bacteria was significantly decreased by pretreatment with 0.5% (p < 0.05) and 3.5% (p < 0.05) MPC polymer, but not by treatment with 0.1% MPC polymer. Based on these results, the inhibitory effects of 3.5% MPC polymer on the adherence of Spn and NTHi were examined.

Five strains of *Streptococcus pneumoniae* (Spn) and nontypeable *Haemophilus influenzae* (NTHi) were investigated, and cells treated with phosphate-buffered saline (PBS) were used as a control. The number of adherent bacteria was determined. When treated with 2-methacryloyloxyethyl phosphorylcholine (MPC) polymer, adherence of both Spn and NTHi was significantly suppressed at 3.5% and 0.5% MPC polymer concentration compared with the control, but bacterial adherence was not suppressed at 0.1% MPC polymer concentration (*p < 0.05). CFU, colony-forming units.

### Effects of MPC polymer and PC-KLH on bacterial adherence in vitro

**Figure 3.**
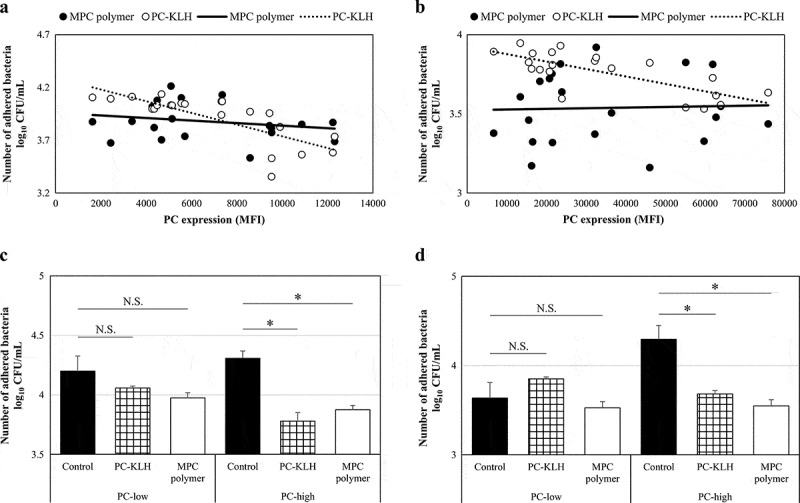
Effects of MPC polymer and PC-KLH on the adherence of Spn and NTHi *in vitro.*

The PC expression and the inhibitory effect on bacterial adherence were compared. The significant negative correlations were observed between PC expression and the number of adhered bacteria for both Spn (r = – 0.78, p = 0.00016) and NTHi (r = – 0.74, p = 0.0061) after pretreatment with PC-KLH (Figure 3(a,b)). However, a negative correlation was not found with MPC polymer (Spn:r = 0.04, p = 0.56; NTHi: r = 0.04, p = 0.56) (Figure 3(a,b)).

The relationship between bacterial phosphorylcholine (PC) expression and the number of adhering bacteria when cells were treated with PC-key hole limpet hemocyanin (PC-KLH) or 2-methacryloyloxyethyl phosphorylcholine (MPC) polymer is shown. (a:*Streptococcus pneumoniae* [Spn], n = 21; b: nontypeable *Haemophilus influenzae* [NTHi], n = 21.) When cells were treated with MPC polymer for both Spn and NTHi, the number of adhering bacteria was similar regardless of the level of PC expression. The cells were treated with MPC polymer or PC-KLH (c and d). Cells treated with phosphate-buffered saline (PBS) were used as the control. Bacterial adherence was significantly suppressed when bacteria were treated with MPC polymer and PC-KLH compared with the control in the PC-high groups but not in the PC-low groups (*p < 0.05). N.S., not significant; CFU, colony-forming units; MFI, mean fluorescence intensity.

According to the median value of MFI, the Spn and NTHi strains were divided into two groups – the PC-low expression (PC-low) and the PC-high expression (PC-high) groups. The mean numbers of adhered Spn and NTHi are shown (Spn; PC-low groups: n = 10, PC-high groups: n = 11) in Figure 3(c,d). In the case of Spn, no inhibitory effect on adherence was observed in the PC-low groups even when treated with MPC polymer (p = 0.12) or PC-KLH (p = 0.14) (Figure 3(c)). However, in the PC-high groups, treatment with MPC polymer (p < 0.01) or PC-KLH (p < 0.01) significantly inhibited adherence (Figure 3(c)). Similarly, in the case of NTHi, the MPC polymer and PC-KLH did not inhibit adherence in the PC-low groups (MPC polymer p = 0.28, PC-KLH p = 0.12) but inhibited adherence in the PC-high groups (MPC polymer p < 0.01, PC-KLH p < 0.01) (Figure 3(c,d)).

### Inhibitory effects of MPC polymer on bacterial adherence in vivo

**Figure 4.**
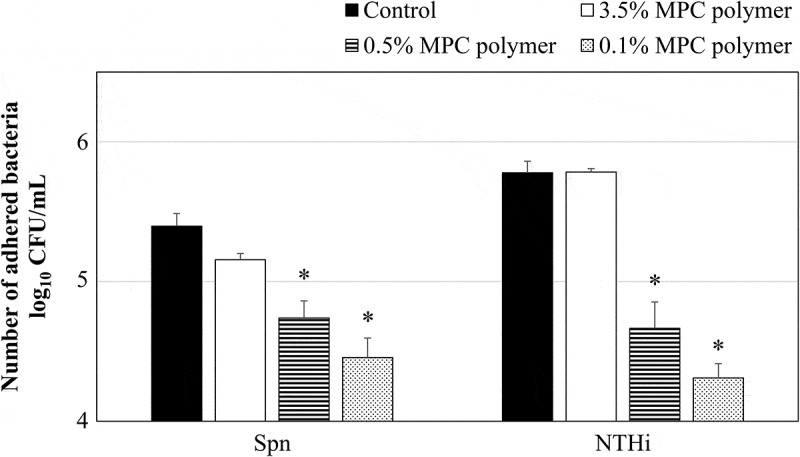
Effects of MPC polymerconcentration on the adherence of Spn and NTHi *in vivo.*

The inhibitory effecton adherence was confirmed using different concentrations of the MPC polymer. The nasal cavities of the mice were pretreated with PBS as a control. As shown in Figure 4, the number of adhered bacteria was significantly decreased by pretreatment with 0.5% (p < 0.05) and 0.1% (p < 0.05) MPC polymer, but not by treatment with 3.5% MPC polymer. Therefore, in this study, the 0.5% MPC polymer was administered to the nasal cavity of the mice.

The number of adhering bacteria when the mouse nasal cavity was treated with phosphate-buffered saline (PBS) was used as the control. When treated with 2-methacryloyloxyethyl phosphorylcholine (MPC) polymer, bacterial adherence was significantly suppressed at ≤0.5% for *Streptococcus pneumoniae* (Spn) and at ≤0.5% for nontypeable *Haemophilus influenzae* (NTHi) compared with control (*p < 0.05). CFU, colony-forming units.

### MPC polymer adherence time in vivo

**Figure 5.**
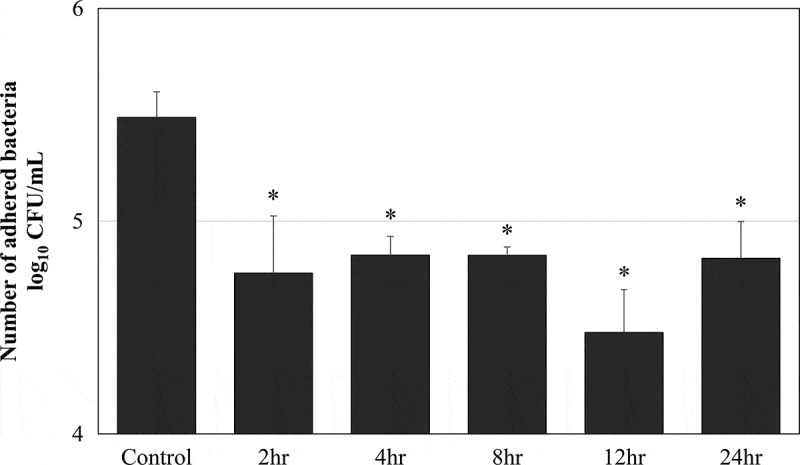
MPC polymer adherence time *in vivo.*

The bacterial adherence time was examined by using five clinical isolates of Spn. The control was the average number of adhered bacteria when treated with PBS at each time point. Treatment with 0.5% MPC polymer significantly inhibited adherence from 2 to 24 h (p < 0.05). The number of adhered bacteria was lowest after 12 h (Figure 5). However, there were no differences in the suppression effect at 2, 4, 8, 12, and 24 h. Therefore, the MPC polymer was administered to the nasal cavity at 12 h.

*Streptococcus pneumoniae* was administered into the nasal cavity of BALB/c mice as a control, and the mean number of bacteria adhering at the indicated times (2, 4, 8, 12, and 24 h after administration) was determined (n = 5). When the nasal cavities of the mice were treated with 2-methacryloyloxyethyl phosphorylcholine (MPC) polymer, bacterial adherence was significantly suppressed for 2 to 24 h there after. The number of adhering bacteria was lowest after 12 h (*p < 0.05). CFU, colony-forming units.

#### *Effects of MPC polymer and PC-KLH on bacterial adherence* in vivo

**Figure 6.**
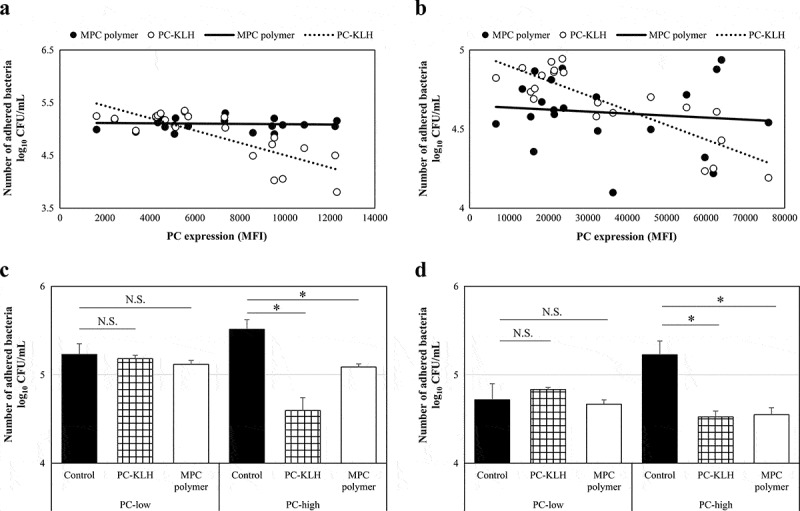
Effects of MPC polymer and PC-KLH on the adherence of Spn and NTHi *in vivo.*

The PC expression and the inhibitory effect on bacterial adherence were compared. The significant negative correlations were observed between PC expression and the number of adhered bacteria for both Spn (r = – 0.07, p = 0.38) and NTHi (r = – 0.85, p = 0.00072) after pretreatment with PC-KLH (Figure 3(a,b)). However, a negative correlation was not found with MPC polymer (Spn :r = – 0.07, p = 0.38; NTHi :r = – 0.12, p = 0.31) (Figure 6(a,b)).

The relationship between bacterial phosphorylcholine (PC) expression and the number of adhering bacteria when cells were treated with 2-methacryloyloxyethyl phosphorylcholine (MPC) polymer and PC-keyhole limpet hemocyanin (PC-KLH) is shown. (a:*Streptococcus pneumoniae* [Spn], n = 21, and b: nontypeable *Haemophilus influenzae* [NTHi], n = 21.) When cells were treated with MPCpolymer for both Spn and NTHi, the number of adhering bacteria was similar, regardless of the level of PC expression. The cells were treated with MPC polymer and PC-KLH (c and d). Cells that were treated with phosphate-buffered saline (PBS) were used as the control. Bacterial adherence was significantly suppressed when treated with MPC polymer and PC-KLH compared with the control in PC-high groups but not in PC-low groups (*p < 0.05). N.S., not significant; CFU, colony-forming units; MFI, mean fluorescence intensity.

Next, according to the median value of MFI, the Spn and NTHi strains were divided into twogroups – as the PC-low expression (PC-low) and the PC-high expression (PC-high) groups. The mean numbers of adhered Spn and NTHi are shown (Spn; PC-low groups: n = 10, PC-high groups: n = 11) in Figure 6(c,d). In the case of Spn, no inhibitory effect on adherence was observed in the PC-low groups even when treated with the MPC polymer (p = 0.12) or PC-KLH (p = 0.14) (Figure 6(c)). However, in the PC-high groups, treatment with the MPC polymer (p < 0.01) or PC-KLH (p < 0.01) significantly inhibited adherence (Figure 6(c)). Similarly, in the case of NTHi, MPC polymer and PC-KLH did not inhibit adherence in the PC-low groups (MPC polymer p = 0.28, PC-KLH p = 0.12), but inhibited adherence in the PC-high groups(MPC polymer p < 0.01, PC-KLH p < 0.01) (Figure 6(c,d)).

#### Effect of MPC polymer on nasal mucosa cells

**Figure 7.**
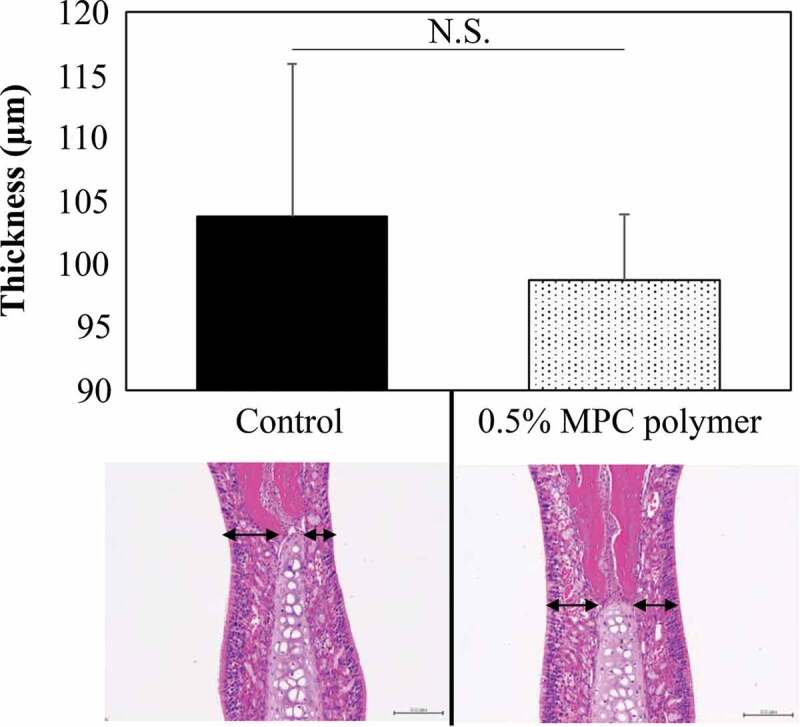
Effect of MPC polymer on nasal mucosa cells.

The effect of intranasal administration of MPC polymer on the nasal mucosa of the mice was evaluated by the mucosal thickness at the nasal septal cartilage site. Nasal cavities administered with PBS were used as a control and compared with nasal cavities treated with MPC polymer. Each study was conducted with three mice, and the histological findings of one mouse are shown in Figure 7. No tissue damage was observed histologically following pretreatment of the nasal mucosa with 0.5% MPC polymer. Although pretreatment with MPC polymer appeared to shrink the nasal mucosa, the thickness of the nasal mucosa did not differ significantly from the control.

The effect of 2-methacryloyloxyethyl phosphorylcholine (MPC) polymer on the mucous membrane of BALB/c mice following administration into the nasal cavity was examined. Phosphate-buffered saline (PBS) administration was used as a control and compared with the administration of 0.5% MPC polymer. The thickness of the mucosa at the upper end of the nasal septal cartilage was measured and averaged. Three mice were used for each group. No change in mucosa thickness was observed after treatment with 0.5% MPC polymer compared with control (upper). When MPC polymer was administered, the cellular structure of the mucosa remained histologically unchanged. N.S., not significant.

## Discussion

MPC is derived from polymerizable methacrylates with a polar phospholipid group on their side chains [16]. The PC group has a phospholipid-like structure that is very close to epithelial cells and can artificially create a surface similar to a cell membrane, making it harmless to the human body [16]. Moreover, MPC polymer effectively suppresses the typical inflammatory reaction of adhered cells [17]. The present study demonstrated that the MPC polymer attached to Detroit 562 cells in a concentration-dependent manner. The reason that MPC coating surface provides a higher contact angle for water is that the hydrophobic alkyl methacrylates-enriched air–material interface reduces the surface free energy [16]. Although MPC polymer is considered to have a high affinity for cells, when 5% MPC polymer was used in our investigations, all the cells peeled from the plate. This phenomenon might be associated with a previous finding that a ≥ 5% concentration of MPC polymer significantly reduced physical and chemical properties [18], suggesting some unknown effect on the cells.

To clarify the association of PC expression with the effects of the MPC polymer, we investigated the relationship between PC expression and the number of adherent bacteria. PC is the ligand of PAF-R, playing a role in bacterial adherence, and is considered one of the virulence factors of Spn and NTHi [19,20]. The present study showed that, in both Spn and NTHi, the numbers of adherentbacteria were negatively correlated with PC expression by pretreatment with PC-KLH. Briles et al. [19] reported that a mouse antibody to PC could protect mice from lethal infection with mouse-virulent human isolates of Spn. In a mouse model of NTHi-induced pulmonary infection, serial passage of NTHi increased both the PC content and the bacterial resistance to pulmonary clearance, whereas no such elevation was observed for NTHi mutants lacking PC [21]. Moreover, we previously reported that the numbers of adhered Spn and NTHi were positively correlated with PC expression [15]. In contrast, the present study showed that treatment with MPC polymer reduced the adherence of Spn and NTHi, regardless of PC expression. In addition, the PC expression of the MPC polymer could not be confirmed (data not shown). The results suggested that the MPC polymer suppressed bacterial adherence regardless of the relationship between bacterial PC expression and cellular PAF-R. As a mechanism of inhibition of bacterial adherence by MPC polymer, Hirota et al. [12] reported that coating coverslips with nonaqueous MPC polymer decreased bacterial adherence and suppressed biofilm formation by the super hydrophilic action of the MPC polymer–coated surface. In addition, bacteria have adhesion factors such as PSaA for Spn [22] and HMW for NTHi [23] on their cell surfaces. The components of these adhesion factors are proteins, and free water on the surface of MPC inhibits protein adsorption and disadheres to cells [24].

We clarified the association of PC expression with the effects of MPC polymer by investigating the effect of inhibition of PAF-R by PC-KLH. In the present study, we showed that the MPC polymer only inhibited the adherence of bacteria in PC-high groups. Spn with high PC expression causes more invasive infection than Spn with low PC expression [19]. Moreover, PC expression in NTHi is associated with prolonged colonization of the nasopharynx inpatients with otitis media with effusion [20]. This suggests that the MPC polymer suppresses the adherence of highly pathogenic Spn and NTHi to cells. There is a report [12] that MPC polymer not only suppresses the increase of resident oral bacteria but also suppresses the increase in the dental caries-causing bacterium *Streptococcus mutans*, which supports our speculation. Because MPC polymer has an inhibitory effect on bacteria that are not covered by the current vaccines, the MPC polymer is considered useful for preventing infection. Moreover, this study showed that the MPC polymer did not inhibit the adherence of bacteria in PC-low groups. This result suggested that the MPC polymer has no effect on low-pathogenic resident bacteria.

We assessed the effect of 0.5% MPC polymer *in vivo*. Spn adherence was ≤0.5% and NTHi adherence was ≤1%, and aninhibitory effect on bacterial adherence was observed. This result suggests that bacterial adherence was suppressed by a concentration of MPC polymer lower than that identified *in vitro*. However, even at ≥12 h after the administration of the MPC polymer to the nasal cavity of the mice, bacterial adherence was inhibited, suggesting that treating nasal mucosal epithelial cells in the body with MPC polymer could exert a long-term effect on bacterial adherence.

The present study observed no histological damage following treatment with the MPC polymer. Although we did not confirm the attachment of the MPC polymer*in vivo*, we speculate that the MPC polymerdoes not injure the nasal mucosa, even when it is attached for a long period. Yumoto et al. reported that MPC polymer treatment reduced subsequent TLR2-mediated innate immune responses and protected oral epithelial cells from chemical irritants [25]. These findings suggest that the MPC polymer is a safe material and does not affect epithelial cells. In fact, MPC has been utilized in various biomedical applications [14], including recent applications in dental materials, such as dental composite materials and dentin-bonding agents [12].

Our study has some limitations. First, the amount of bacteria used was low. However, we believe that the universality of the results is maintained because we used the samples collected from various sites. The second limitation is pertaining to the method used for measuring the number of bacteria: the average of the measurements conducted by two individuals was used to determine the number of bacteria. Becausethe measurements were conducted in the state wherein the PC expression was unknown, it was considered that the bias regarding PC expression and bacterial adherence was eliminated. The third limitation is pertaining to the epithelial cells used. Although the use of human normal epithelial cells may be more clinically useful, these cells hamper the growth of Detroit 562 cells in culture. Nevertheless, we used a pharyngeal cancer-derived cell line, and because it is of human origin, the results of this study were not considered different from when normal cells were used.

In conclusion, MPC polymer inhibits the adherence of Spn and NTHi, regardless of PC expression on the bacterial surface. Furthermore, it was found that MPC polymer inhibits adherence of highly pathogenic bacteria and does not affect less pathogenic bacteria. There was no effect on nasal mucosal cells. These findings suggest that the MPC polymer could be applied in topical medications, such as gargles and nebulizers, and a new effect can be achieved by suppressing the adherence of highly pathogenic bacteria.

In the future, we intend to proceed with clinical research regarding the application of the MPC polymer in topical medications.

## Data Availability

The authors confirm that the data supporting the findings of this study are available within the article.
